# Drs. Gall and Spurzheim on the Structure of the Brain

**Published:** 1817-08

**Authors:** 


### *Drs. Gall and Spurzheim on the Structure of the Brain.*

( Continued from page 53. )

Having, in our last Number, mentioned the cerebellum as originating from the corpora restiformia, it is now in order that we should state the progress of its gradual developement. These corpora restiformia are situated on each side, and towards the posterior surface of the medulla oblongata; from them a continuous fasciculus of medullary fibres may be easily traced to the cerebellum. This, indeed, was known to the older anatomists, who called it "processus cerebelli ad medullam oblongatam." In its course upwards, it meets with a mass or ganglion called corpus dentatum, which contains much brown matter, and is considered by Dr. S. a preparatory apparatus, destined to increase the medullary filaments which enter it. From this the primary fasciculus receives, as usual, accessions of white nervous bundles, and, thus strengthened, divides into many medullary branches and layers, which form the hemispheres of the cerebellum. One of these branches or layers goes to form the processus vermiformis, which is considered the fundamental or primitive portion of the cerebellum from its being found in birds and some fishes, whose cerebella have no lateral lobes. In this manner is evolved and perfected the mass of the cerebellum. From the above views, it would appear that it, like the brain-proper, is constituted chiefly of one great order of fibres; viz. the *diverging.*

A second order, however, still remains to be spoken of; viz. the *converging* or *uniting* fibres, and it now behoves us to explain Dr. S's views with regard to them.

These begin on the surface of all the convolutions, and are derived from the brown matter (here properly enough called *cortical),* which covers their outer surface. In the cerebrum, they converge from all parts, towards the mesial line, and thus form the corpus callosum, the fornix, and numerous other commissures; all of which, to the sight, are evidently fibrous: here they mix or unite with the corresponding converging fasciculi of the opposite hemisphere, and thus produce a communion or reciprocation of influence, between the two sides of this important organ, destined for similar functions. In the inferior cornua of the lateral ventricles, these converging fibres may be readily seen *decussating* with the diverging series that come from the corpora striata.

In the cerebellum, a similar order of fibres springs from the grey substance on its surface, and these converge on each side, towards the middle line, where they unite, to form the tuberosity of the pons Varolii. The pons, then, is the great commissure of the cerebellum, and forms the superincumbent stratum of transverse fibres which, we before mentioned, must be removed, before we can see the subjacent longitudinal fibres of the pyramids passing upwards underneath. The size of the pons, from this cause, is always in proportion to the lateral parts or hemispheres of the cerebellum; and in some of the lower class of animals, which have no cerebellum, save the primitive part, called processus vermiformis, we invariably find there is no tuber or pons.

A very new idea still remains, and one of the greatest importance, as far as regards the pathology of hydrencephalus. The diverging and converging fibres necessarily cross and intersect each other at the bottom or root of the convolutions, and form a tissue which may be called the *ceiling* of the lateral ventricles. The strength of this tissue is not inconsiderable in the healthy state; but when its resistance is overcome, in the dead subject, by the fingers introduced into the ventricles, or in the living body by the gently increased pressure of water collected and accumulating in these cavities, the convolutions are easily separable in the middle line, and can be unfolded without destruction or laceration of their substance, like the sleeve or fold of a gown after the stitches that retain it are cut. Hence, it is apparent, that each convolution is a sort of cul-de-sac or small canal, and not a uniform substance; that it consists of a portion of cerebral matter, folded on itself, having cortical substance without, and medullary or white matter within. The folds may be said to be in a state of most intimate contact rather than of absolute cohesion; since, when we make a section of one of the convolutions, and direct a stream of air from a blow-pipe, or water from a syringe, upon the cut part, the separation (even if we try all we can to the contrary) will take place in the middle line very readily, and no where else; and the separated surfaces will be found with a smooth, and as it were polished, appearance, which could never follow simple laceration of the part. What was a convolution is thus converted into a thin membranous-like expansion, with a layer of brown matter externally, and white internally. This Dr. S. affirms to be the actual morbid change produced in every case of chronic hydrencephalus of any size or standing; that in such cases the convolutions are obliterated, and thence the relative situation of cerebral parts greatly altered; but that, in effect, there is never any *absorption* of the brain.

There is nothing throughout the whole province of anatomy and pathology, that has given rise to more various speculations, than the use of the ganglia interspersed throughout the nervous system. We have not space to detail the conjectures offered by *Winslow*, *Willis*, *Vieussenius*, *Lancisi*, *Mecker*, *Zinn*, *Monro*, and *Scarpa*; and it is the less necessary, as they are generally known to most readers. We have only, therefore, to state simply the idea of Drs. Gall and Spurzheim on this point, an idea which seems to be exclusively their own. They conceive, that the ganglia spread over the whole body, are small masses of cineritious or cortical matter, which certain nerves pass through, and where these nerves are strengthened, in the same manner as the pedunculi of the brain are increased in the thalami optici and corpora striata.

Dr. S's manner of procedure consists in beginning with the decussation at the medulla oblongata, and tracing the diverging fibres upwards, in the order of their direction, as far as the convolutions. He then traces the converging order of fibres according to their direction, from the convolutions to the middle line. Thus he begins his demonstrations with the medulla oblongata, and ends it with the convolutions and commissures. In the course of it, he seldom *cuts* with the scalpel, but merely employs its *handle wetted*, to remove the superincumbent parts, in order that the fibres, and their direction, may be distinctly seen. He always scrapes *across* the course of the fibres, lest it should be said that they are the creation of the handle of the knife drawn along the pulpy parenchyma of the brain.

He reprobates the horizontal slicing of the brain, which is the mode so long and so universally employed in demonstrating this organ; and alledges that by this method, not only its structure cannot be seen, but that the relative situation and connection of the parts, are destroyed by it. Would it not be absurd to *chop off* the extremities in transverse pieces, or to make horizontal sections of the trunk of the body, in order to shew the muscles, blood-vessels, and nerves of the limbs, or the situation and connection of the thoracic and abdominal viscera? Yet absurd as this supposed practice may appear, a similar mode has been hitherto adopted in demonstrations of the brain.

We have now finished the outline we proposed to give of Drs. Gall and Spurzheim's meritorious labours in this field. It is quite general, and does not pretend, on minor points, to be absolutely correct. We have not taken our notions from hearsay or verbal descriptions, but have several times witnessed, with the closest attention, Dr. S's demonstrations of the *recent unprepared* brain; and can vouch for the truly satisfactory, as well as able, manner in which they are performed. We have also repeated, in private, the dissections after his manner; and the result has been a belief of their entire correctness. If we have so far succeeded as to kindle in our Readers any interest for these investigations, we refer them to the large work and plates, published by the anatomists in question, several years ago, and to the Article " Cerveau" in the *French* Dictionary of Medical Science.

Let us now run over the contents of Dr. Gordon's book, and examine in detail, the various objections he has urged against the views of which we have given a synoptical account.

In his Introduction, we are told that the parts of the brain are extremely intricate and numerous, and therefore that an acquaintance with its structure is exceedingly difficult of acquisition; in the next sentence, however, he consoles the ignorant and the lazy, by telling them that such knowledge is of " no essential consequence in the practice of medicine;" that " diseases are distinguished from each other, and proper remedies applied to them by skilful and eminent practitioners, who are satisfied, and justly so, with a general view of this organ." Such opinions, held forth in print by a *public teacher* of *anatomy*, appear to us peculiarly ominous, and we feel it our bounden duty to mark them with our severest reprehension. We should be indeed sorry ever to see it tried as an experiment, with how little real and accurate knowledge our profession can be practised. We will not pretend to inquire into the skill or eminence of those practitioners of whom he speaks, because we hope that his allegation is unfounded in fact; but if any thing we say can have weight with the younger class of our readers, we would earnestly exhort them to build the foundation of their professional skill on the firm and prosperous soil of accurate anatomical and pathological knowledge, not of the brain merely, but of every part of the body, and, in their dreams of future eminence, to covet no laurels which have not buried their roots deep in this honourable though arduous field. Of such laurels the verdure is perpetual; and they will bloom with undying freshness around the tomb and the ashes of him who shall win and wear them, while the manœuvres by which local repute is sometimes extended, and eminence acquired, have perished with those transient circumstances to which they owed their petty sum of success.

After a few pages, written in rather a querulous spirit, all about Gall and Spurzheim, and the attention with which their investigations have been honoured, "improperly," of course, the author introduces a long quotation from *Malpighi's* Exercitatio Epistolica, another from *Vieussenius*, and others from *Leeuenhoeck*, *Haller*, *Mayer*, *Reil*, *Cuvier*, and *Portal*, all intended to prove that the fibrous structure of the brain was known to these eminent anatomists. Yet, after liberally praising these authors, a few pages afterwards he turns quickly round, and, with amazing inconsistency, declares, that the fibrous structure of the brain is "merely hypothetical;" that we do not see actual fibres, but merely "a fibrous appearance."—Now we appeal to our readers, if this is not arrant hypercriticism, the acme of quibbling. A fibrous *appearance* truly! Why, we only know objects in nature by their appearance; into their occult essences our faculties cannot penetrate. But perhaps Dr. G. is a disciple of the Hudibrastic school, and

> "Can reduce all things to *acts*,
> And know their nature by *abstracts*;
> Where entity and quiddity,
> The ghost of defunct bodies, fly."

Surely, in every anatomical question, the evidence of the sense of sight must be admitted, and its decision must be ultimate. To this sense the fibres of the brain are perfectly apparent, without the aid of the microscope; what occasion then is there for refining? At this rate, anatomical investigations must be stopped *in limine*; and in the end, the sum of all our knowledge will be that nothing is known.

At p. 30, the author adverts to the use or function which Drs. G. and S. have assigned to the grey matter, viz. of forming the white. This he calls a mere hypothesis, and dismisses it at once with the epithet of "absurd." Now, on this point we would suggest a few observations.

These zealous anatomists having observed that the fœtal brain contains a much larger share of brown matter, in proportion to the white, than that of the adult; having observed also, that in the adult, wherever the white fibres are increased or reinforced, brown matter is invariably there present, in quantity corresponding to that increase; and moreover, that in the ganglia dispersed throughout the nervous system, brown matter is always contained, and that the nerves entering into these ganglia go out enlarged in size *in proportion* to the quantity of that brown matter: observing these facts throughout the whole range of animals that have a nervous system, they adopted the opinion that the brown substance is accessary to the formation of the white, and called it the "matiere nourriciere" of the latter substance. It has also been called the "matrix" of the white; but the latter term suggests a disputable idea, and is, in our opinion, an instance of unhappy analogy: let us attend, however, to their facts, and from them estimate the probability of their inference. When we find it as a law in the animal œconomy, that the white and brown nervous matter are in a determinate proportion or relation to each other; that wherever brown matter exists, there accessions of white medullary fibres take place, and no where else; and that when a small nerve imbeds itself in the brown matter of a ganglion, it goes out from it enlarged; it surely appears a pretty legitimate induction that the brown matter is the medium from which the white is formed. All organized parts are formed ultimately, of course, from the blood, but proximately from some other substance. Thus the fibres of bone, though secreted from the blood, are formed proximately from cartilage; the nails from a white, glairy, tenacious, albuminous-like matter deposited at their roots; and the substance by which solutions of continuity from wound are repaired, is formed from the layer of coagulable lymph exuded from the divided vessels.

Thus also the fluid secretions are not formed from the blood without the aid of an intermediate organ or substance. Urine, bile, saliva, or gastric juice, though certainly derived ultimately from that fluid, owe their proximate origin to the kidnies, liver, salivary glands, or stomach. It is not sufficient ground to reject an opinion simply because we do not, in the present state of our knowledge, understand its rationale, or how it is so. What could appear *a priori* more improbable than the received chemical doctrine of *disposing affinity*? According to this doctrine, when two bodies have no tendency to combine, the addition of a third body, which has a strong affinity for the compound, often causes them to do so. Criticism may say that this theory is "absurd," because it supposes a body, not yet formed, to be possessed of affinities. The phænomenon, notwithstanding, is true, however contradictory it may appear to our limited faculties.

We are certain, that food taken into the stomach becomes blood, but how it does so we know not: this blood, again, is accessary to the growth and nutrition of every part of the body; but of the steps of the process, in either case, we are ignorant; we see only the result. This branch of physiological inquiry has been gradually advancing, until it has reached the confines of utter darkness: at these confines it must be content to wait, until some gifted genius of the first order shall arise, capable of illuminating the path, and carrying it farther. Meanwhile we do not see why the new opinion, that the grey matter forms the white, should be treated with so much abruptness and disrespect. The mind, when it invariably sees any two phænomena constantly conjoined in time and place, as in the present case, is habitually led to consider and call that relation cause and effect. This, we are persuaded, is a just account of the idea of causation, disengaged from its metaphysical attire and its scholastic envelopements; for this idea is the result of observation and not of intuition. Upon the whole, therefore, if the pre-existence of the brown matter to white in the fœtal brain, and the other circumstances of proportion already stated, should be established and more fully ascertained by future extensive observation, we shall think the opinion, founded upon such observation, that the one causes the formation of the other, to be duly proved. In the mean time, while it is brought forward as a speculation by Drs. Gall and Spurzhiem, we think it apparently as much supported by facts as the other theories of any vital process, and we presume these gentlemen only mean to bespeak for it a parallel portion of consideration.

Dr. Gordon thinks that it would be "quite as physiological" to conceive that the function of the brown matter is "to *absorb* the white." Now this conception must take it for granted, that the different fibrous parts of the brain originate from above, and verge downwards to the medulla oblongata, diminishing in size (like an inverted cone) wherever they meet brown matter. But we cannot admit this *absorbing* function, unless it were as physiological to conceive that the nerves of smell, sight, taste and hearing, originate in the nose, eye, tongue or ear, and proceed inwards, as to believe, which we are old-fashioned enough to do, that they originate in the brain and medulla oblongata, and are destined or distributed to the external organs whose functions they execute. Nor can we admit what this conception takes for granted, namely, the formation of the cerebral mass from above downwards, seeing that this is not the order of Nature in the developement of those parts; for the spinal marrow and medulla oblongata are without doubt the primitive part of the nervous system, as they are found not only in acephalous fœtuses, but in that class of the lower animals which have no brain or cerebellum. As we advance in the scale of being and of intellect, we observe cerebral parts superadded to these elementary nervous masses. Indeed, it would appear that the brain is only subsidiary, or, as it were, an appendage to the spinal marrow, and that the former is the instrument solely of the intellectual faculties, thought and volition. On this subject the experiments of Le Gallois are most illustrative. Now, although the nervous influence undoubtedly proceeds both upwards to the brain from the spinal marrow, and downwards (during the operations of voluntary motion) from that organ to the spinal marrow and inferior nerves,* yet, in the scale of creation, the order of formation of the brain un-

* For the proof of this see the Experiments of Le Gallois and Dr. Wilson Philip.

questionably is from below upwards, and its parts are successively evolved in that direction.* This, therefore, is quite incompatible with Dr. Gordon's gratuitous supposition, and with the conclusion he would deduce from it, that the brown matter absorbs the white.

But farther—respiration is generally considered to be the cause of animal heat; yet, there are some who hold it "as physiological" to believe (with the immortal Harvey) that the function of this process is to *cool* the blood, and to dissipate the heat formed in the body by some *other* cause. Yet this contrariety of opinion cannot invalidate the *law*, deduced from the observation of many phænomena, that there is in all warm-blooded animals an evident connection or relation between the respiration and the degree of their temperature. In the case before us, therefore, whatever diversity of opinion may exist, whether, in short, the brown matter serves to nourish and increase, or to absorb and destroy the white, (granting, for the sake of argument, that either supposition is "as physiological" as the other) yet the *fact* still remains impregnable to cavilling, that these two kinds of matter always exist in definite and determinate proportions to each other; and we conceive that Drs. Gall and Spurzheim have great merit in having been the first to point out this as a *law* in the nervous system.

From page 38 to 61, the author comments upon Dr. Spurzheim's description and demonstration of the cerebellum, which he objects to *in toto*. Here he is very angry at Dr. S. for having, it seems, in one of his public demonstrations at Edinburgh, read passages out of a System of Anatomy which, it would appear, Dr. Gordon has published. In this system, as well as at page 264 of the 49th Number of "The Edinburgh Review," Dr. G. had said that the corpus dentatum of the cerebellum contains no brown matter. Spurzheim, however, displayed that body in the recent brain, compared it with his opponent's descriptions, and, in the opinion of the audience, (as the other confesses) fixed upon him more than a suspicion of inaccuracy. Now, we admit, that it was very impertinent on the part of Spurzheim to be guilty of *laese-majesté* towards Dr. Gordon, in his own lecture room, where he was, so to speak, perched on the pedestal of his own fame; moreover, it was very *mal-apropos*, particularly

---

* *See* Mr. Abernethy's second lecture on Mr. Hunter's Theory of Life, where this subject is eloquently and admirably treated.

before so large and respectable an assembly; and we do think that our learned author has a right to feel sore—yea, very sore—on so touching an occasion. However, he may console himself; his is not the only anatomical book that cannot bear to be contrasted with Nature.

From page 61 to 135, the author is employed in objecting to the description of the two orders of fibres, diverging and converging. In this part, we think there is greater asperity, not to say coarseness of expression, than in any other. It seems to be part of his tactics, to bepraise Reil in the same proportion that he bespatters Spurzheim. Our limits will not afford room for an extract; but should any of our readers have a longing to see the true "*kali causticum*" of criticism, we would recommend them to gratify their curiosity by looking into this part of the work.

We conceived, that if there was in the views and demonstrations of which we have so often spoken, one part more impregnable than another, it was that about the pathology of hydrencephalus, and the unfolding and obliteration of the convolutions. Yet the views on this point, though strengthened by the high authority of *Morgagni*, he combats, from p. 135 to 163; and, as usual, there is not a word of truth in them. Opposition is always acrimonious, in proportion as it is unreasonable.

From page 163 to 178, he criticises the plates; *of course*, they are "indistinct," "inaccurate," "unnatural," "imaginary," "fictitious," &c. &c. He then comments on the procedure which Dr. S. adopts in demonstrating the brain, and though he cannot deny it some share of merit, he asserts it to be "a rude method," and is still an advocate for the old mode of transverse slicing and paring. Now we certainly say, that the brain should be examined in all possible ways; but we also say, that it is impossible to trace the direction of its fibres, or the connection of its different parts, by the method of transverse sections, practised by Vicq. d'Azyr, and adopted in our schools. We have no objection, however, to that mode being continued, provided such laborious trifling do not arrogate to itself the name of science, and the praises of utility. We know some worthy persons that choose to employ their time in painting the colours of a leaf, or the hues of a shell; and others, again, that take pleasure in impaling butterflies upon pins, and making drawings of them for the inspection of the curious. Why not, then, cross-cut, slice, pare, and paint the brain? It will be a species of *delassement*; and we can see no reason why men should not have the privilege of spending, or rather mis-spending, *their own* time according to their own will and pleasure.

At the conclusion, Dr. Gordon indulges in a peroration, in which the more loose and scattered rays of censure are duly collected and concentrated. Indeed, in respect of censure, we are of opinion, that throughout the whole treatise, he has exceeded to such a degree as must defeat his own purpose. Probably, he would have been much more readily believed, if he had mixed "a thread of candour" with the web of his controversial discussion. Surely, the Doctor is not so far gone in the art of authorship, without having learnt the *finesse* of "damning by faint praise." In its present state, however, his whole performance smacks strongly of prejudice and a pre-determination to reject; so much so, that the candid reader, as he goes on, is induced to besprinkle all his allegations *cum grano salis*.

He finally takes leave of the reader, by telling him he is "determined not to resume the subject."—*What a pity!*—Now we do not say this in jest, for it gives us real pleasure always to foresee plenty of occupation in the way of our critical calling: like skilful sportsmen, we know what is *game*, and are happy to see it *multiply*. But, even, on other grounds, we should not be displeased to see Dr. Gordon resume the subject: he has much still to controvert; and discussions, if properly conducted, will always be productive of good—he will be sure, at any rate, to prove *some person* in the wrong. Yet, notwithstanding the weight of these reasons, we have heard many persons say (persons, of course, naturally very dull, and of no taste) that they did not regret the Doctor's present determination to silence, as they had no wish to be treated with a second volume of captious remark, peevish irritability, and heavy compilation from older authors; moreover, (though we are sure they had no pretensions to the gift of prophecy) they ventured to foretell that what he has already written will be but short-lived,—that its days are numbered, and that it will soon be consigned "to lie in cold obstruction and to rot," like the mortal remains of those former anatomists from whose learned labours he has so liberally quoted.*

* Though our author's book is full of the praises of anatomists, both dead and living, and quotations from their works, he could not find room

We are willing to exalt, rather than depreciate, the researches of the older anatomists; yet, while we are anxious to apportion merit to whom merit is due, we think, that to allege that they were acquainted with, and had accurately described the fibrous structure of the brain *throughout*, and that they have anticipated Drs. Gall and Spurzheim in all their discoveries, is to assert a great deal too much—more indeed than the truth.—Whatever had been discovered or described by former authors, in any respect analogous to the demonstrations in question, had been subsequently, for the most part, doubted or denied, and eventually forgotten: they were, at least, quite unknown to much the greater part of the profession of the present day. The investigations even of *Reil*, the latest of those authors whom Dr. Gordon quotes, and on whose descriptive Essays, in "Gren's Journal" for 1795, and in "The Archives of Physiology," he lays so much stress, were scarcely known beyond the little Germanic circle in which he lived. But that even *he* anticipated Gall will by no means be believed; for it is known, that prior to 1795, the latter had commenced his researches in the dissection of the brain; in fact, his physiological inquiries have occupied him more or less from his boy-hood, and his investigation of the structure of the brain followed up the first dawn of his physiological opinions *pari passu*. His peculiar mode of dissection, and the outline of his anatomical and pathological views, were known in Vienna for a number of years before he began to visit the different cities of Europe. How shall it be said, then, that he was anticipated by *Reil*? That he has availed himself of the labours of preceding anatomists where they were correct, needs not be denied; he would have been culpable had he not so done. Purifying their descriptions from error, and improving upon what they had less perfectly performed, is the task which he has executed, and it is a task for which few were qualified. Both he and Spurzheim constantly repeat that they do not claim the discovery of many new facts. Their great merit, in our opinion, consists in the connection which they have been the first to establish between the facts already known; and in the general conclusions which they have formed and deduced from them.

---

to take the slightest notice of the accurate and truly classical work of *Dr. Monro Secundus*, on the Brain and Nervous System!—*There are reasons for every thing*—Συνετοισι.

Besides having introduced, or at least revived, a better mode of dissecting the brain, they are unquestionably the first who have pointed out the certainty of the decussation, the successive reinforcement of the longitudinal fibres in the pons, crura, optic thalami, and corpora striata; the two sets of fibres in the brain, and the generality of the commissures; (which last seems to assist in explaining the unity of action of the two hemispheres, and of the nerves of each side.) Moreover, they have been the first to point out the small size of the cerebellum in the fœtus, compared with that of the brain-proper; and that betwixt infancy and puberty, the former increases its size in a much greater ratio than the latter. They have also pointed out to future observation, that the frontal sinuses only exist in old age, at which period there takes place an absorption of the diploë, with somewhat of a diminution of bulk of the encephalon. They have also observed that there is a general tendency of the bony cavity of the head to lessen itself upon its contents in old age; hence the frequency of exostosis from the inner table of the skull, and mania arising therefrom, at this period of life rather than at any other.*

Likewise, they have been the first to shew the swellings in the spinal cord of the calf; the proportion between the brown and white matter throughout the brain, spinal marrow, and ganglia; and, (what, perhaps, is their most meritorious discovery, as it is most exclusively their own) they have been the first to ascertain the true *origin* (to use the common language) of the pretended cerebral nerves: We now know that the nervi optici do not arise from the thalami, but from the nates; and that the 3d, 4th, 5th, 6th, 7th, 8th, &c. arise from certain points in the *medulla oblongata*. With regard to the origin assigned by them to these nerves, Dr. Gordon has nothing to object, an evident proof that his researches in the writings of the ancients had produced nothing to his purpose. Instead, however, of allowing them any merit in this department, he has cautiously abstained from once alluding to the subject! As his censure is so indiscriminate, we should

---

* That the skull becomes thin in old age by absorption, is maintained also in the "Anatomia Senilis," published in 1799.—*Tenon* published in "The Memoirs of the Institute" for the year 6, a paper on the human cranium: from his observations, it appears that, in old age, it loses *two-fifths* of its weight.

think it incumbent upon him to *prove* that it is merited also on this important point. For him to assert, in general terms, that all these things were known before, is an assertion contradicted by plain facts. Such views, if they had been clearly and connectedly detailed by preceding inquirers, would have been taught as a matter of course in our schools of anatomy; yet they were not. Dr. Gordon himself has, we believe, been a public lecturer on anatomy for a number of years, and we challenge him to prove that these views, or any thing analogous to them, were heretofore taught by him, well informed as he *now* appears to be, of the discoveries of the older anatomists, and familiar as he *now* is with the researches of *Reil*. We can answer for it he taught none such.—Nay, we firmly believe, that it was only since he buckled on his armour for this Controversy, that Reil has been ransacked.

The outline we formerly gave, and what we have just said about the originality of Drs. Gall and Spurzheim's labours, will enable our readers to form a just estimate of their deserts. We are far, however, from thinking their views of the brain complete; since, so far as we can see, they give no account of the pituitary gland, the infundibulum or the pineal gland, and its pedunculi. But the lover of science will not be fastidious, or niggardly of his approbation, as if

> "Nil actum reputans, si quid superesset agendum;"

He will rather wonder that these anatomists have done so much, than that they have much still to do; and will be satisfied that they have opened up a route, which may at last conduct us to a much more complete knowledge of the functions, as well as of the structure of the brain and nerves.

We have not time, nor limits, to enter more at large into the nature of Dr. Gordon's objections; indeed, it would be almost useless, for, after all, the controversy cannot be settled on paper: it is in the anatomical theatre, and in the *palæstra* of actual demonstration of the *recent unprepared* brain, that the contest is to be decided. Thither Dr. Spurzheim challenges his antagonists, trusting in the greatness of his cause's strength, and in the fidelity of his observations. Meanwhile, he has answered his opponent's objections as satisfactorily as *mere writing* can do, in the pamphlet noticed at the beginning of this article. In that pamphlet also, he has succeeded, almost beyond hope, in pointing out much inaccuracy, inconsistency, and contradiction in the various written attacks of this *Goliath* of *northern* criticism: he has made his own opinions, and very expressions, sit in judgment upon him, and has finally "taken his own sword and slain him therewith." He uses towards him throughout, a freedom of style, and a tone of reply, which one can see have been provoked by the unhandsome, and often indecent, conduct of his adversary. Indeed, we cannot read this pamphlet without perceiving that the behaviour of Dr. Gordon has been most illiberal and most unscience-like. We have heard, on this point, many particulars recited, which we were slow to believe, until they came to us from quarters which imply the impossibility of misrepresentation. These, however, we forbear to communicate to our readers, because we have a true respect for the literary character, however abused, and are ever more ready to conceal and compassionate its frailties and aberrations, than to exaggerate and expose them.

Dr. Spurzheim may, however, abstain from publishing any future remarks; he may well rest satisfied with the approbation of his labours, which many eminent anatomists and pathologists have shewn. Dr. Parry* is with him, if Dr. Gordon is against him; and doubtless, might we venture an opinion, "*L'un vaut bien l'autre.*"

But, after all, Dr. G's opposition cannot, in the end, do any manner of harm. His work, now before us, is neither more nor less than an operose commentary on the Report of the Committee of the French Institute. That learned body, very properly, nine years ago, thought it incumbent upon them, as the depositaries of science, to receive, with great caution, views so novel as those of Drs. Gall and Spurzheim. They started doubts very freely, and referred largely to the writings of preceding anatomists. Dr. Gordon has scarcely quoted an author besides those mentioned in their Report. The doubts of the Committee have been exaggerated, and their objections magnified in his hands, but the measure of praise, which they awarded as justly due, he has invariably and care-

---

* See Elements of Pathology and Therapeutics, p. 225, et seq.

In common with the whole enlightened part of the Profession, we deeply lament that stroke of disease which threatens to deprive medical science of the high talents and powerful mind of Dr. *Parry*. Without intending any thing like a miserable pun on such an occasion, we may well say, "Quando ullum invenient *Parem*?"

fully suppressed. Surely, he must have been under the influence of strong resentment when he stooped to such disingenuousness as this — disingenuousness most reprehensible in any man, but more peculiarly unbecoming the character which ought to be cultivated by a lover and teacher of science.

Of the *physiological* opinions of Drs. Gall and Spurzheim we can hardly speak, without, in some degree, going out of our way: yet on them, also, we may be permitted a remark or two. The real nature of their investigations has been greatly misrepresented by some, and imperfectly understood by all. In common with every *enlightened* physiologist of the present day, they hold that the brain is the material medium of the immaterial mind; seeing, in this lower world, spirit does not, and cannot, act or manifest itself, excepting through the instrumentality of organic matter. From this datum, which is almost self-evident, they proceed to inquire whether the *totality* of the brain acts in executing any *individual* operation of intellect; or whether *each* mental power has a *separate* and determinate portion of the brain assigned as its instrument. They have adopted the latter opinion, and believe, that though the cerebral substance is unique, each portion of it executes a special function, in the same manner as there is one nervous fasciculus for sight, another for hearing, smelling, &c. though all these fasciculi consist, as far as we can judge, of similar nervous matter.

Thus explained, the scope of their inquiries is perfectly legitimate; but that they have already succeeded in ascertaining what particular portions of brain are destined to the manifestation of particular faculties, passions, or feelings, is to us by no means proved. To use a familiar form of speech, "it is too good news to be true!" Their opinions, though illustrated with great force and acuteness of observation, have not yet received the sanction of general experience; and although, solely for the latter reason, it would be unwise to deride or reject them altogether, yet we must suspend our opinion, not from any want of respect for Drs. G. and S's talents or veracity as observers of Nature, but until inductions are multiplied *by others,* so as to produce greater probability, if indeed this can ever be the case.

Mr. John Hunter, many years ago, endeavoured to estimate the degree of intelligence in the different classes of animals, by comparing the size of their brain with that of their spinal marrow. It is apparent, that the investigations of that illustrious individual were founded on the same radical principle with those of the gentlemen we have so often mentioned; viz. that the brain is exclusively the organ of thought, volition, and the other powers of the mind.

Before concluding, we must turn our attention to the investigations of Drs. Gall and Spurzheim, in the 49th Number of "The Edinburgh Review," on purpose to mark it with most merited reprobation. It is an article unworthy of science—unworthy every way of the writer. Every man endowed with common feelings, however hostile his opinions may be to those attacked, will view that attack, such as it is, as one of the most uncandid libels that ever stained the whiteness of paper; and will consider that it almost deserves other than mere literary censure. In this noted article, facts are distorted, feelings are wounded, and private character traduced,—and all with an "*Am not I in sport?*" It is written in a loose, *tranchant,* gossiping, *fanfaronading* style, in which smartness is made to supply the place of argument, and abuse to serve in lieu of demonstration; just, forsooth! as if observations founded on the sure basis of nature were to be razed by a repartee, or put down by the pop-gun of ridicule. After all, however, the writer is much more successful at *railing* than *raillery,* and at *wrangling* than *reasoning*. Indeed, had not common fame told us to the contrary, we should have supposed the article to have been penned by some erudite *spinster,* who gratified at once her knack of scribbling and scandal; or some *amateur-anatomist,* who must needs write on things he did not sufficiently understand.

We strongly deprecate the use of such a tone and manner in scientific discussions. Even the impunity which is, in a manner, secured to such injurious critiques, only serves, in our opinion, to aggravate their culpability. For our part, we unaffectedly admire the mild and correct idea of the moralist of antiquity,—"vir probus, etiam impunitate propositâ, abstinet injuriâ."

If towards Dr. Gordon we have appeared severe in aught, let not our motives be misunderstood. With him, personally, we are totally unacquainted; and as to his literary character, we do not deny that he has superior talents. But we are of opinion, that good-fortune, or something else, has egregiously spoiled both them and him; nay, the very luxuriancy of his genius seems to have operated to its own detriment, having grown, as it were, to seed, and become thereby comparatively useless. In some such way only, can we account for his always writing like an *enfant gatè*, and assuming, in his criticisms, a tone of unjust contempt for others, and of confidence in his own powers, equally unjust.* Excess of self-approbation is, doubtless, as incompatible with good writing as with good breeding; it fosters a regardless, forward deportment, to the entire destruction of that literary chastity, which adds grace to the writer, while it modestly wins the affections of the reader; that intellectual delicacy which (as *Boileau* hath it,)

> "Plait a tout le monde, et ne sauroit se plaire."—Sat. 2.

By this time, however, we believe, he bitterly repents his temerity, and is sensible, when too late, that he has committed an act of suicide on his own fame. The harm he has done himself is incalculable; and really, though the mischief is of his own making, we cannot but relent, and feel for him.

Here, however, our limits require we should stop. In our Review of this singular Controversy, we have been guided by the sole wish of rendering truth triumphant, and of asserting the predominancy of genius, over the prescriptions of authority. The party against whom we have decided is a veritable *Apollo triformis,* and in his triple capacity of reviewer, author, and teacher of anatomy, is not a little formidable to us,—but *n'importe*—we care not what resentments we shall brave, when duty requires it of us. It shall ever be our pleasure to pay homage to science in whomsoever found, or by whomsoever opposed; to sustain truth against the dogmas of mere prejudice; to look oppression in the face and reprove it; and, on all occasions, to be strenuous supporters of the natural aristocracy of talents and merit.

---

* For proof of this, see the Analysis in "The Edinburgh Review" of Mr. Abernethy's Lectures on Mr. Hunter's Theory of Life; the Analysis in the same of Sir Everard Home's Paper; and others of the same stamp.

